# Loosening Identification of Multi-Bolt Connections Based on Wavelet Transform and ResNet-50 Convolutional Neural Network

**DOI:** 10.3390/s22186825

**Published:** 2022-09-09

**Authors:** Xiao-Xue Li, Dan Li, Wei-Xin Ren, Jun-Shu Zhang

**Affiliations:** 1 Department of Civil Engineering, Hefei University of Technology, Hefei 230009, China; 2 School of Civil Engineering, Southeast University, Nanjing 211189, China; 3 College of Civil and Transportation Engineering, Shenzhen University, Shenzhen 518061, China; 4 Key Laboratory for Resilient Infrastructures of Coastal Cities, Ministry of Education, Shenzhen University, Shenzhen 518061, China

**Keywords:** multi-bolt loosening, loosening identification, vibro-acoustic modulation, time-frequency diagram, convolutional neural network

## Abstract

A high-strength bolt connection is the key component of large-scale steel structures. Bolt loosening and preload loss during operation can reduce the load-carrying capacity, safety, and durability of the structures. In order to detect loosening damage in multi-bolt connections of large-scale civil engineering structures, we proposed a multi-bolt loosening identification method based on time-frequency diagrams and a convolutional neural network (CNN) using vi-bro-acoustic modulation (VAM) signals. Continuous wavelet transform was employed to obtain the time-frequency diagrams of VAM signals as the features. Afterward, the CNN model was trained to identify the multi-bolt loosening conditions from the raw time-frequency diagrams intelligently. It helps to get rid of the dependence on traditional manual selection of simplex and ineffective damage index and to eliminate the influence of operational noise of structures on the identification accuracy. A laboratory test was carried out on bolted connection specimens with four high-strength bolts of different degrees of loosening. The effects of different excitations, CNN models, and dataset sizes were investigated. We found that the ResNet-50 CNN model taking time-frequency diagrams of the hammer excited VAM signals, as the input had better performance in identifying the loosened bolts with various degrees of loosening at different positions. The results indicate that the proposed multi-bolt loosening identification method based on VAM and ResNet-50 CNN can identify bolt loosening with a reasonable accuracy, computational efficiency, and robustness.

## 1. Introduction

A high-strength bolt connection is the key component of large-scale steel structures. The behavior of the bolt connection under loadings directly affects the load-carrying capacity, safety, and durability of the whole structure. The preload force of bolt tightening directly determines the clamping force between the two connecting parts. The insufficient preload force inevitably leads to the loosening of the connecting bolt and the loosening of the connecting parts. High-strength bolt connections are complex in the steel structure because they are the main force transmission component, and there are many factors affecting the force performance of high-strength bolt connections. Under the action of serving the environment multi-physical field coupling (e.g., vehicle load, wind load, temperature load, atmospheric corrosion), the steel structure joints are damaged (e.g., through multi-bolt loosening). This leads to structural load-bearing capacity reduction and even over-all structural failure. It is therefore necessary to identify the bolt loosening and evaluate the safety status of high-strength bolt connections. Modern high technologies such as big data and artificial intelligence provide an intelligent way to ensure the safety of inspectors, save costs, and improve the accuracy and efficiency of the loosening identification of bolt connections.

Nondestructive testing methods are commonly used in the detection of loosening bolt connections [1]. They can be divided into vibration, electromechanical impedance, acoustic emission, ultrasound, machine vision, and (intelligent) percussion methods. The vibration method [2] selects hammer excitation or a vibration generator and other methods to excite the high-strength bolt connections and then uses accelerometers to collect vibration response signals for identification. The advantages of the vibration method are mainly its ease of operation and low cost, while its disadvantages consist of its low accuracy, low sensitivity, dependence on operator experience, and significant influence by environmental noise, which translate to poor robustness. Ultrasound methods mainly include active sensing methods, acoustoelastic effect methods, and nonlinear ultrasound methods. Nonlinear ultrasonic methods mainly include harmonic methods, such as the subharmonic method [3] and second/high-order harmonic method [4], and modulation methods, such as the percussion-acoustic modulation method [5] and the vibro-acoustic modulation (VAM) method [6]. The nonlinear ultrasonic method relies on contact sensors, while the VAM method is sensitive to contact-type defects and has the advantages of high accuracy and sensitivity. The VAM method can achieve early identification of damage compared with other nonlinear identification methods, and the test equipment is simple and convenient. Amerini et al. [7] found that the modulation edge frequency amplitude increased with the increase of high-strength bolt loosening by using VAM techniques for the identification of conventional bolted structures. Their results indicate a good correspondence between the theoretical analysis and experimental results. Jaques et al. [8] found that the VAM technique could be used for the identification of high-strength multi-bolt loosening in aircraft structures, as the VAM satisfied the requirements of insensitivity to shape identification and high detection efficiency. This technique was used to develop a high-strength multi-bolt tightness inspection method without a reference datum and was applied to the inspection of bolted structures on satellite aluminum plates [9].

The current applications of various bolt loosening identification methods are individual. These methods include wave-based ones, such as wave or ultrasonic waves, and vibration-based ones. These methods have individual advantages and disadvantages in terms of accuracy, sensitivity, robustness, cost, and requirements for inspectors. Both types of methods essentially process the acquired signal; however, the difference lies in the frequency band of the signal of interest. Various wave-based identification methods are concerned with signals in the high-frequency (HF) band, whose frequency is generally in the range of kHz or even 10,000 Hz or more. These methods belong to the local damage of bolt loosening identification methods. Alternatively, vibration-based identification methods are concerned with signals in the low frequency (LF) band, whose frequency is generally in the range of kHz or ≤100 Hz. These methods belong to the overall damage of bolt loosening identification methods. In this respect, existing methods for identifying concealed damage in multi-bolt connections ignore the information in the LF band or the HF band, and the information used is incomplete. Thus, an effective combination of both methods can provide a more efficient method of loosening the identification of multi-bolt connections.

The multi-bolt connection loosening of steel structure is not only hidden but also has an unusually complex status. The complex status includes the location of the loosening occurrence, the degree of the loosening, and the evolutionary pattern, which are exceptionally complex. This complexity can be solved using a suitable pattern recognition method. For example, Wang et al. [10] proposed a new strategy based on the acoustic emission technique to detect bolt looseness, and this research represented the first attempt to identify multi-bolt looseness via the AE-based method. Using a deep convolutional neural network (CNN) to solve engineering problems has become a research hotspot in many fields recently [11,12,13,14,15,16]. Wang et al. [17] proposed a new robotic-assisted active sensing method based on our newly designed PZT-enabled smart gloves and position-based visual serving technique. Tan et al. [18] proposed a method based on continuous Bayesian Networks that can perform a safety evaluation on truss bridges. Zhuo et al. [19] proposed a multi-bolt loosening method based on the support vector machine classification of sound signals. It enables the identification of various environmental noise signals and sound signals of multi-bolts with different locations and loosening degrees. Zhao et al. [20] proposed a bolt loosening angle identification technique by combining deep learning and machine vision. In recent years, the deep CNN has risen rapidly; it has a more powerful learning capability than traditional pattern recognition methods and can provide a new solution to the problem of the intelligent identification of complex diseases in steel bolt connections. With the development of sensing technology and signal processing methods [6,10,21,22,23,24], the advantages of wavelet transform and other time-frequency analysis methods have also been illuminated, and include strong computing power and efficient data analysis capabilities. To date, target recognition techniques based on big data and deep learning have been successfully applied to the identification and localization of loosened bolt connections [20,25,26]. Li et al. [27] proposed a novel method based on an unthresholded assembled recurrence distance matrix and multi-label CNN for structural loosening identification under nonstationary ex-citations. Li et al. [28] further a proposed acoustic emission wave classification method based on synchrosqueezed wavelet transform, and the results show that the multi-branch CNN could identify surface rail cracks, where both impact-induced and crack-propagation-induced acoustic emission waves were identified. Yu et al. [29] proposed a novel method based on deep convolutional neural networks to identify and localize damages of building structures equipped with smart control devices. Furthermore, a vision-based crack diagnosis method is developed using deep convolutional neural network and enhanced chicken swarm algorithm [30]. Deep learning techniques rely heavily on data-driven feature extraction by learning a large number of samples.

A loosening identification method of high-strength multi-bolt connections based on VAM signals is proposed in this paper. It takes multi-bolt connection structures under the joint action of HF ultrasound and LF excitation. For the hidden and unusually complex state of the steel high-strength bolt connections structure, such as that of large steel bridges, the signal issued by multi-bolt loosening on bridges has contact nonlinear characteristics. Moreover, through adding the white Gaussian noise (WGN) into original multi-channel signals, the Noise-Assisted MEMD [31,32] was proposed to reduce mode mixing problem effectively. Considering the serious interference of noise to the engineering structure in the outdoor area, WGN Random noise is added to the HF signal during the test. The amplitude of the percussion was random while the random white noise was added. This can also eliminate the impact of operating noise, which is one of the important purposes of this study. Time-frequency diagrams with edge-frequency components from wavelet transform are obtained through modulating the HF vibration and LF vibration. Then, combining the diagrams with the ResNet-50 CNN, we effectively extract the characteristics of high-strength multi-bolt loosening by using the CNN model, which exploits the advantages of wavelet time-frequency analysis in processing non-smooth signals and the powerful image classification ability of ResNet-50 fully. Afterward, we achieved loosening identification based on VAM signals of high-strength multi-bolt loosening.

## 2. Loosening Identification Method for High-Strength Multi-Bolt Connections

### 2.1. VAM

**Figure 1:**
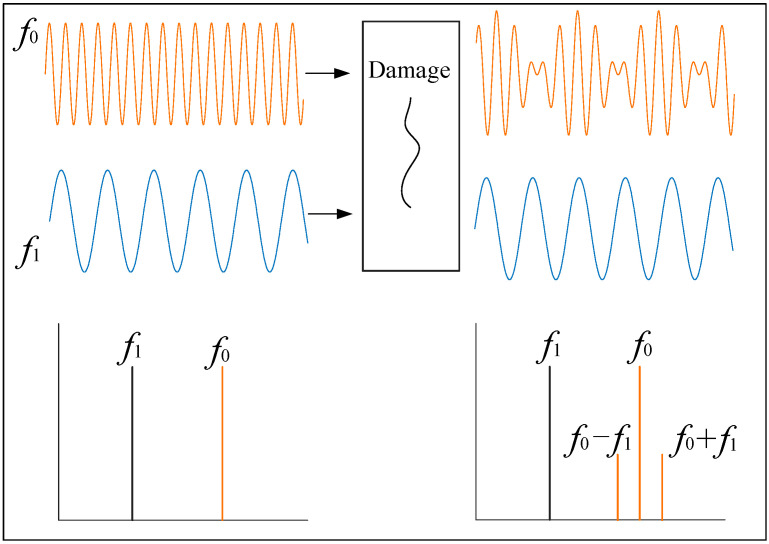
Principle of vibro-acoustic modulation (VAM) technique.

The VAM technique is a nonlinear acoustic identification method. The damage can be detected because of the different stresses at different moments under LF vibration excitation, and the amplitude or phase of the HF signal passing through this interface there-fore changes (modulation). The principle of VAM is shown in Figure 1. The LF vibration signal (*f*_1_) and the HF ultrasonic signal (*f*_0_) are simultaneously applied to the specimen in the test, and if there are defects such as cracks in the specimen, the received signal spectrum contains the edge-frequency (*f*_0_ ± *nf*_1_) components; otherwise, *f*_0_ and *f*_1_ have no interaction, and the received signal spectrum is the same as that of the input signal [33,34,35]. Therefore, the quality of the tested parts can be evaluated by monitoring the presence or absence and amplitude of the modulated edge-frequency components. However, the identification of sidebands is difficult to separate under the influence of multi-bolus structure modulation and noise [36], and is highly susceptible to different factors, which limits the efficiency and application of sideband extraction [37]. The CNN selected for this study has powerful image classification, target identification, and image segmentation capabilities. The CNN is more sensitive to deep, specific nonlinear feature representations and can accurately distinguish between different structures and sideband components under noise. It performs efficient and accurate extraction of nonlinear damage features, which leads to effective identification of multi-bolt preload loss.

### 2.2. Wavelet Transform

The continuous wavelet transform is performed on the vibroacoustic modulated signal, where the continuous wavelet transform is defined as the convolution of the signal *x(t)* with the complex conjugate $\psi^{*}$ of the wavelet basis function:

(1) $$WT\left(a,b\right)=\frac{1}{\sqrt{a}}\overset{\infty }{\underset{-\infty }{\int }}x\left(t\right)\psi^{*}\left(\frac{t-b}{a}\right)dt$$

where the wavelet basis function $\psi^{*}$ is a Complex Morlet wavelet. Complex Morlet is a commonly used complex-valued wavelet with good resolution in time-frequency domains compared with other wavelets. It reflects good time-frequency aggregation in time-frequency diagrams, making it suitable for processing non-smooth sound signals. The expression of the Complex Morlet in the time domain is as follows:

(2) $$\psi^{*}\left(t\right)=e^{j\omega_{0}t}e^{-\frac{t^{2}}{2}},\;$$

where $\omega_{0}$ is a center frequency of the wavelet function.

Then, the continuous wavelet transform of the vibroacoustic modulated signal is generated as a time-frequency diagram, using the functions in the MATLAB wavelet toolbox.

### 2.3. ResNet-50 CNN Model

**Figure 2:**
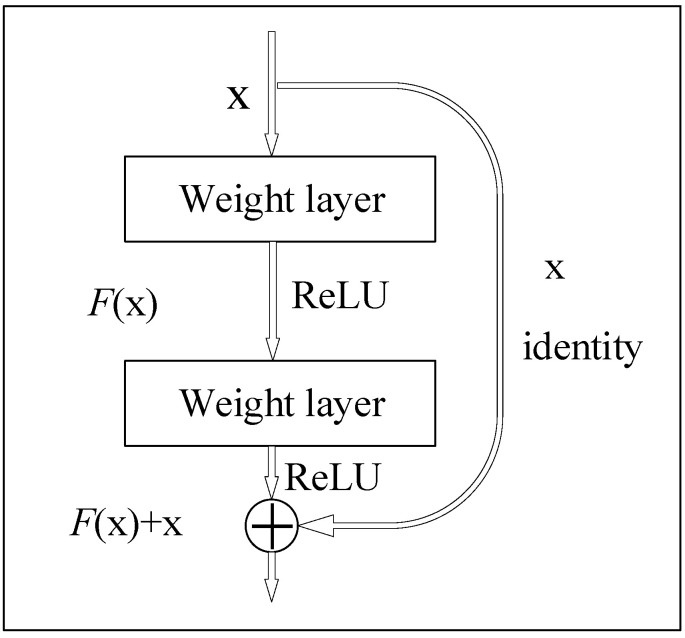
ResNet-50 residual block.

**Figure 3:**
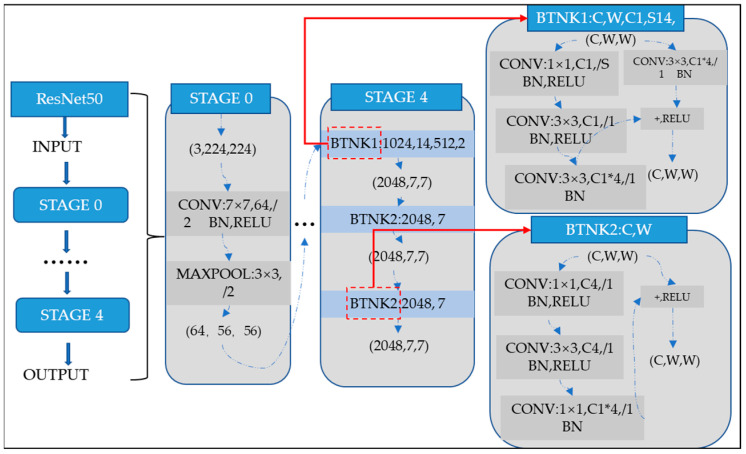
ResNet-50 neural network configuration.

The CNN is a class of feedforward neural networks that includes convolutional computation and has a deep structure. It is good at processing multi-dimensional data such as images and is one of the representative algorithms of deep learning. The convolutional layer, pooling layer, and fully connected layer make up a typical CNN model. In addition, auxiliary layers such as the activation layer and random deactivation layer are included in the CNN for improving the generalization ability and learning performance, which prevent overfitting. The convolutional layer performs a convolutional operation on the input using a series of convolutional kernels with learnable parameters and then generates a series of feature diagrams by sequentially sliding local receptive fields over the input. The feature diagram consists of a number of neurons, called units. Each cell in the feature diagram of the current convolutional layer is connected to a local region in the feature diagram of the previous layer by a set of weights and an activation function. The output ($X_{j}^{l})$ of the *l*th feature diagram of the *j*th convolutional layer can be calculated using the following equation:

(3) $$x_{J}^{l}=f\left(\sum_{i\in M_{j}}x_{i}^{l-1}*k_{ij}^{l}+b_{j}^{l}\right),\;$$

where $k_{ij}^{l}$ represents the weight matrix of the jth convolution kernel, $b_{j}^{l}$ is the corresponding bias matrix, $M_{j}$ is the set of feature diagrams, * denotes the convolution operation, and $f\left(\underset{i\in M_{j}}{\sum }x_{i}^{l-1}*k_{ij}^{l}+b_{j}^{l}\right)$ is the activation function. The pooling layer downsamples the feature diagram output from the convolution layer to reduce the dimensionality of the feature diagram and the number of parameters. This ensures translation invariance and improves the robustness of the model. The maximum pooling operation in the l pooling layer is as follows:

(4) $$x_{J}^{l}=f\left(\beta_{j}^{l}down\left(x_{j}^{l-1}\right)+b_{j}^{l}\right),\;$$

where $down\left(x_{j}^{l-1}\right)$ denotes the maximum downsampling function, while $\beta_{j}^{l}$ and $b_{j}^{l}$ are the multiplicative and additive biases, respectively. Clearly, the convolution layer identifies the local nonlinear features of the previous layer, while the pooling layer fuses similar nonlinear features and removes unnecessary details. The learned feature diagram can be expanded into a vector and classified when it uses a fully connected layer on top of the CNN. The output ($x^{l}$) of the lth fully connected layer is calculated as follows:

(5) $$x^{l}=f\left(\omega^{l}x^{l-1}+b^{l}\right),\;$$

where $\omega^{l}$ and $b^{l}$ are the corresponding weights and biases, respectively. The ResNet [38] CNN model was first proposed by He et al., from Microsoft Research in 2015. Many scholars have studied it deeply since it was proposed. It has gradually become a popular CNN model because of its unique and innovative network idea. Moreover, its CNN is simple in construction, has fewer parameters, and has a remarkable effect. As a result, it is widely used in image segmentation, image identification, and other fields. The general perception is that the deeper the CNN and the more parameters the CNN has, the better the CNN‘s ability to represent the nonlinear structure for traditional neural networks. The CNN generally becomes more effective as the CNN depth increases. However, the problem of gradient explosion or gradient disappearance occurs during the training process of the CNN when the depth of the CNN layers increases to a certain level. This leads to a decrease in the accuracy of the CNN model inversely. The ResNet-50 is a CNN model proposed for the problem of gradient disappearance and gradient explosion. The main idea of ResNet-50 is to improve the traditional CNN by using residual blocks that are connected in a “shortcut connection” arrangement [39]. The nonlinear activation function (ReLU) is used to improve training efficiency and avoid gradient disappearance. The module of residual blocks is shown in Figure 2. The output of the upper CNN layer is partially saved to the later CNN layers using residual blocks, which effectively alleviate the pressure of learning parameters in the deeper CNN layers. The ResNet-50 CNN model with residual blocks effectively reduces the effects of gradient explosion and gradient disappearance caused by the increasing network depth. The ResNet-50 CNN model can also save more information for learning during training by overlaying the original nonlinear features with the nonlinear features learned from the residual blocks. The CNN performance is superior. ResNet-50 has become a popular CNN model in recent years owing to its few CNN parameters and superior CNN performance. Therefore, the ResNet-50 model is used in this paper, and its CNN structure is shown in Figure 3.

Some hyperparameters, such as the learning rate, batch size, and the number of epochs, are not trainable but obviously affect the performance of the CNN model. Manual tuning of hyperparameters is a difficult undertaking. Usually, random search and Bayesian optimization are needed to tune the hyperparameters automatically. This study aimed to obtain the optimal set of these hyperparameters by the Bayesian optimization algorithm. Bayesian optimization is an informed search. It utilizes the performance of the parameters that have been searched before to speculate how to perform better later. This makes the search space smaller and the search more efficient. Bayesian optimization algorithm constructs a probability proxy model based on the prior knowledge of the sampling points, and then iterates continuously to increase the amount of information. The prior knowledge is modified and updated to obtain the optimal value of the objective function with less time cost. Its essence is to update prior knowledge according to new information, thus generating posterior knowledge.

The hyperparameters of the CNN models, including learning rate, batch size, and the number of epochs, were tuned with the help of Bayesian optimization algorithm and the validation data set. A validation dataset needs to be constructed when Bayesian optimization is applicated, and the objective function that needs to be minimized is the validation error $E^{V}\left(\bar{\theta }\right)$, where $\bar{\theta }$ is the vector of considered hyperparameters [40]. It can be assumed that the objective function is taken from a Gaussian process prior, as in

$E^{V}\sim N\left(0,K\right)$ with

(6) $$K=\left[\begin{array}{ccc} k\left({\bar{\theta }}_{1},{\bar{\theta }}_{1}\right) & \cdots & k\left({\bar{\theta }}_{1},{\bar{\theta }}_{n}\right)\\ \vdots & \ddots & \vdots \\ k\left({\bar{\theta }}_{n},{\bar{\theta }}_{1}\right) & \cdots & k\left({\bar{\theta }}_{n},{\bar{\theta }}_{n}\right) \end{array}\right]+\sigma_{noise}^{2}I,$$

where $\sigma_{noise}$ is the standard deviation of Gaussian noise, *n* is the number of iterations, and *k* is the covariance function, which is the automatic relevance determination Matérn 5/2 kernel in this paper [41]. Under the Gaussian process prior, $E_{1:n}^{V}$ and $E_{n+1}^{V}$ are jointly Gaussian, and the predictive distribution can occur based on the previous observations, $D_{1:n}=\left\{{\bar{\theta }}_{1:n},E_{1:n}^{V}\right\}$:

(7) $$\begin{array}{c} E_{n+1}^{V}D_{1:n}\sim N\left(\mu \left({\bar{\theta }}_{n+1}\right),\sigma^{2}\left({\bar{\theta }}_{n+1}\right)+\sigma_{noise}^{2}\right),\\ \mathrm{with}\{\begin{array}{c} \mu \left({\bar{\theta }}_{n+1}\right)=k^{T}{\left(K+\sigma_{noise}^{2}I\right)}^{-1}E_{1:t}^{V}\\ \sigma^{2}\left({\bar{\theta }}_{n+1}\right)=k\left({\bar{\theta }}_{n+1},{\bar{\theta }}_{n+1}\right)-k^{T}{\left(K+\sigma_{noise}^{2}I\right)}^{-1}k\\ k={\left[k\left({\bar{\theta }}_{n+1},{\bar{\theta }}_{1}\right)\cdots \left({\bar{\theta }}_{n+1},{\bar{\theta }}_{n}\right)\right]}^{T} \end{array} \end{array}$$

Then, the next point $\left({\bar{\theta }}_{n+1}\right)$ is evaluated by acquiring the function that is constructed from the predictive posterior distribution. Using the expected improvement over the best expected value $u_{best}=argmin_{{\bar{\theta }}_{j}\in {\bar{\theta }}_{1:n}}\mu \left({\bar{\theta }}_{j}\right)$, it has a closed form solution with the Gaussian assumption:

(8) $$\begin{array}{c} a_{EI}\left({\bar{\theta }}_{n+1}\right)=\sigma \left({\bar{\theta }}_{n+1}\right)\left[Z\Phi \left(Z\right)+\emptyset \left(Z\right)\right]\;\mathrm{with}\\ Z=\frac{u_{best}-\mu \left({\bar{\theta }}_{n+1}\right)}{\sigma \left({\bar{\theta }}_{n+1}\right)}, \end{array}$$

where Φ and ∅ are the cumulative distribution function and probability density function, respectively. The next point to evaluate can be obtained by the maximization of the acquisition function ${\bar{\theta }}_{n+1}={\mathrm{argmax}}_{{\bar{\theta }}_{n+1}}a_{EI}\left({\bar{\theta }}_{n+1}\right)$ [41].

### 2.4. Multi-Bolt Loosening Identification Based on Wavelet Transform and the ResNet-50 CNN

**Figure 4:**
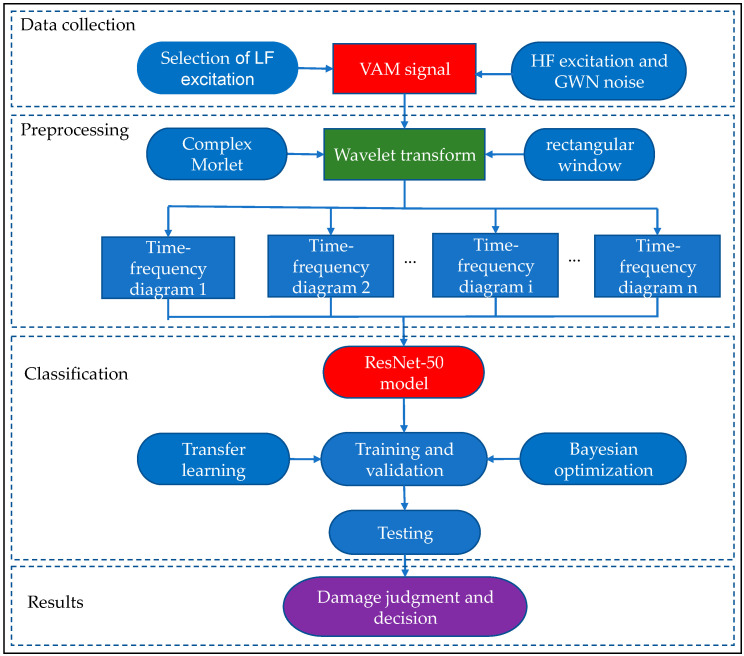
Multi-bolt loosening identification flowchart.

The flowchart of multi-bolt loosening and preload loss identification based on a wavelet time-frequency diagram with ResNet-50 is shown in Figure 4. This method is divided into two stages: training and testing. The training stage uses a data acquisition instrument to collect a sufficient number of VAM signals for multi-bolt loosening and preload loss. VAM signals are extracted by the signal processing method and then inputting it to the time-frequency diagram obtained by wavelet transform of VAM signals in the CNN. The nonlinear feature extraction is completed on the feature diagrams generated by the convolution operation, and ResNet-50 with set parameters for training is used to obtain a high-strength multi-bolt preload loss recognition model. The test phase uses the trained model to identify the input VAM signal. In order to save time for training and to realize a more robust CNN using limited VAM data available in this study, a pre-trained ResNet-50 is used for transfer learning. Transfer learning is based on previously acquired knowledge to perform the current classification task. It enables the knowledge obtained from previous tasks to be obtained from a different big data source. The Bayesian optimization algorithm is used to tune the hyperparameters. The last three fully connected layers of the model full are replaced in this study, which are the connected layer, SoftMax layer, and classification layer. The output size of the full connected layer is modified to 16, while other parameters remain unchanged.

## 3. Experimental Verification

### 3.1. VAM Test

**Figure 5:**
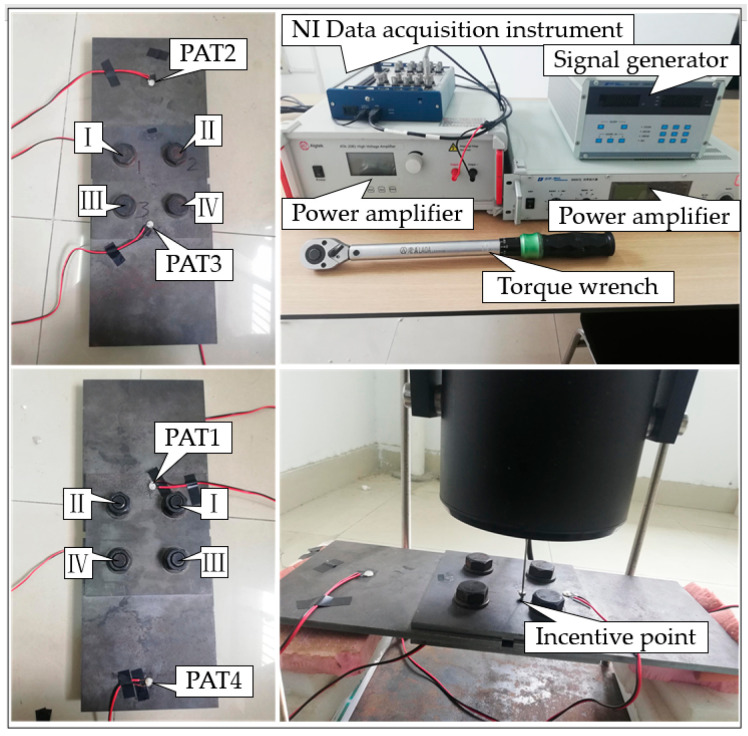
Experimental setup.

Four steel plates were connected using four M20 high-strength bolts to form a test specimen, as shown in Figure 5. The size of the splicing plate was 180 × 180 × 8 mm, the core plate size was 240 × 190 × 8 mm, the materials were Q345 steel, and the four high-strength bolts were numbered Ⅰ, Ⅱ, Ⅲ, and Ⅳ, for easy classification of the CNN identification results. To avoid the occurrence of repeated working conditions due to the symmetry of the piezoelectric ceramic piece, we pasted the four PZT sensors into position, as shown in Figure 5. PZT1, on the same side of high-strength bolt head, is the sensor for emitting signals; PZT2, PZT3, and PZT4, on the same side of the nut, are sensors for receiving VAM signals. The initial torque of the general M20 high-strength bolt is 221 N·m, and the final torque range is 374 N·m to 510 N·m. Wang et al. [6] proved that the bolt can be loosened at an early stage when the pre-tightening force is 50 N·m to 70 N·m, so 70 N·m was selected as the tightening state torque value in order to make this study applicable to more working conditions. The torque wrench used in this test was a pre-set torque wrench with a range of 28 N·m to 210 N·m. To prevent complete fall, we took the minimum working range of the torque wrench as loosening state. So, the starting torque for bolt loosening was selected as 28 N·m. When the torque is other values, it would be assigned according to the principle of proximity.

**Figure 6:**
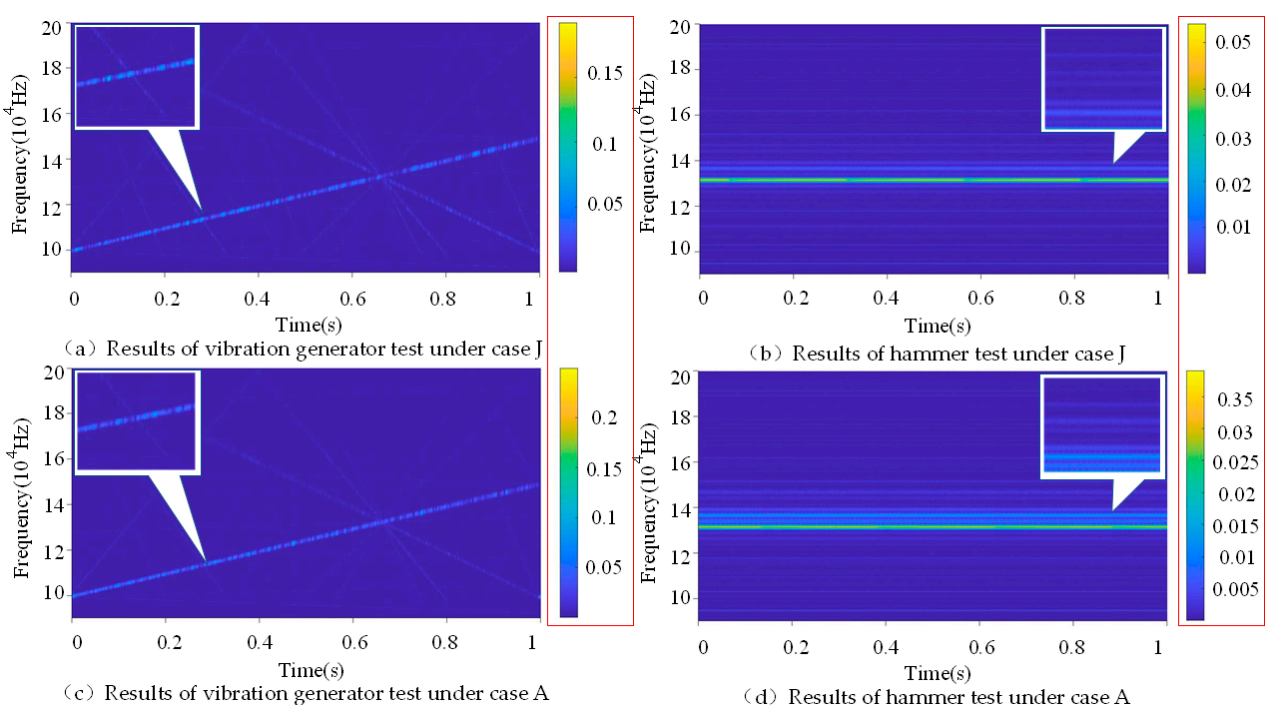
Time-frequency diagram obtained using different test methods excitation frequencies: (**a**) results of vibration generator test under case J; (**b**) results of hammer test under case J; (**c**) results of vibration generator test under case A; (**d**) results of hammer test under case A.

The data acquisition system of this experiment is composed of a vibration generator, NI data acquisition instrument, HF ultrasonic signal generation equipment, and computer, where the LF excitation is generated by the vibration generator or force hammer, and the point of action is shown in Figure 5. The role of the HF signal is similar to that of the carrier in VAM identification, which is used to carry nonlinear modulation information. Generally, the continuous single-frequency sine or sweep signal with a frequency ranging from tens to hundreds of kHz is used. Its amplitude is tens of volts. In order to increase the randomness of the samples, we programmed the random noise and HF excitation by LabVIEW to act on the specimen through PZT. Two different LF excitation forms were selected to compare the effects of different waveforms of LF excitation on the model recognition effect, and a test of the LF excitation form was completed using the vibration generator, where the vibration generator output frequency was 640 Hz. The frequency range of 10–15 kHz linear frequency modulation was the PZT input HF frequency. Another test of LF excitation was completed using the hammer, and the hammer strike frequency was 4 Hz. The frequency of inputted sine wave for PZT was 13.284 kHz. The two tests are referred to as the vibration generator test and the hammer test in the following section. Bolt loosening has obvious contact nonlinear features, but the corresponding time-frequency diagram changes very slightly, and it is difficult to select manually. Thus, deep learning relies on data-driven feature extraction, which can identify the contact nonlinear features of bolt loosening. The wavelet time-frequency diagrams obtained from the two tests are shown in Figure 6. The variation of signal frequency components with time is shown in the wavelet time-frequency diagrams. The warm and cold colors reflect the amount of energy carried by each frequency component of the signal in the wavelet time-frequency diagrams; the warmer its color, the greater its energy. Case J means four bolts are “Tightness”, and case A is all “Loosening”. From Figure 6, not only the energy bar changes during the bolt state transformation, but also the time-frequency characteristics change. When the bolt transfers from tightening to relaxation, it can be seen from Figure 6a,c that the side lobe components on both sides of the signal obtained by the vibration generator test change. Similarly, the side lobe components on both sides of the signal obtained from the hammer test in Figure 6b,d also change.

The test steps are as follows:

Step 1: Simulation of loosening working conditions. The 16 working conditions simulated by loosening different positions and different numbers of high-strength bolts are shown in Table 1, with a high-strength bolt torque of 70 N·m in the tight condition and 28 N-m in the loose condition.

Step 2: Model excitation. The vibration generator test was conducted with a DH40200 vibration generator, and the hammer test was conducted with an LC02 force hammer.

Step 3: Signal acquisition. Sixteen operating conditions were acquired using an NI data acquisition instrument with a sampling frequency of 40 kHz.

### 3.2. Dataset Construction

**Figure 7:**
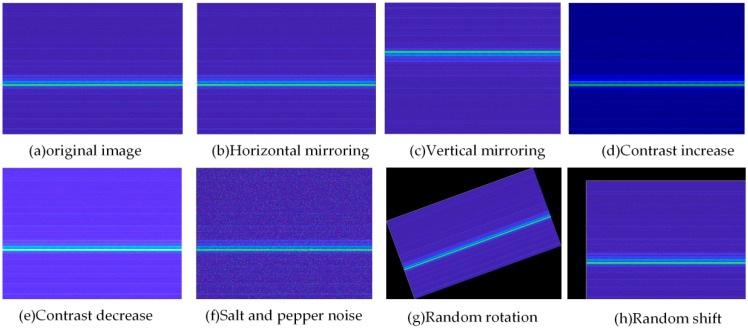
Image augmentation.

Algorithms, computing power, and datasets are the essential triumvirate of big data. The ResNet-50 CNN model was chosen as the algorithm in this paper, and the computing power, computer platform for model running and programming environment are shown in Table 2. For the dataset construction in this paper, the wavelet time-frequency diagrams obtained from the analysis of the VAM signals of 16 operating conditions were categorized and labeled. Each dataset was randomly divided into 500 samples in the ratio of 6:2:2 as the training dataset to train the model parameters. Then, 160 samples were used as the validation dataset to tune the parameters of the model and initially evaluate the accuracy of the model. The remaining 160 samples were used as the test dataset to evaluate the generalization ability of the model. The final constructed dataset, therefore, consisted of 13,120 samples, of which 8000 samples were in the training dataset, and 2560 samples each were in the validation and test dataset. When comparing the effects of datasets of different sizes on the training results, the original sample was extended to 104,960 samples by inversion, translation, scaling, and adding noise and contrast enhancement. Moreover, the corresponding datasets were randomly divided into a training dataset, validation dataset, and test dataset, according in the ratio of 6:2:2. The image augmentation is shown in Figure 7. 

## 4. Results and Discussion

Hyperparameter selection is difficult and time-consuming in deep learning training, as the optimal combination of hyperparameters depends not only on the model itself but also on the software and hardware environment. The hyperparameters of the CNN models, including learning rate, batch size, and the number of epochs, were tuned with the help of Bayesian optimization algorithm and the validation data set. When we used the ResNet-50 model for training, the training and validation datasets were randomly divided into several batches by the batch training method. The training batch (Minibatch) size was 32, the round (Epoch) was 30, the iterations per round was 216, and the total number of iterations was 6480. Each 50 iterations triggered one verification, and the initial learning rate was set to 0.001. The weight decay value was 1 × 10^−5^. The output size was 16, and the damage function used the mean square error. The Stochastic gradient descent with momentum (SGDm) optimizer with a momentum of 0.9 was used. The monitoring index was the average absolute error. The Bayesian optimization algorithm was used to tune the hyperparameters. We set the Dropout layer random dropout probability to 0.4, meaning that 40% of nodes were randomly dropped to further prevent overfitting. Other initial parameter weights for model training were used with the weight values trained on the ImageNet dataset.

### 4.1. Comparison of Different CNN Models

**Figure 8:**
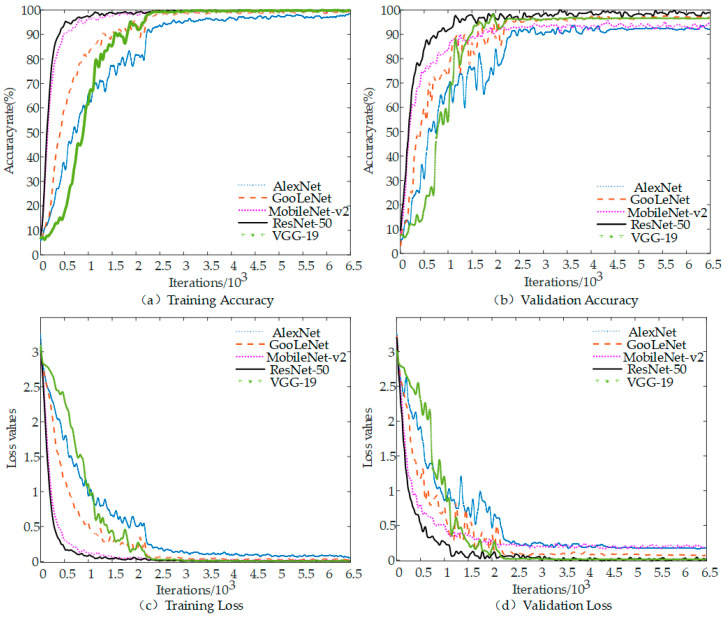
Comparison of training results between ResNet-50 and other CNN models: (**a**) training accuracy; (**b**) validation accuracy; (**c**) training loss; and (**d**) validation loss.

In order to verify the effectiveness and superiority of this research method, we randomly selected AlexNet, VGG-19, GoogLeNet, and MobileNet-v2 classical models for comparison with the training results of the ResNet-50 model. The samples used were obtained from the force hammer experiment. Five model training cycles and validation were performed under the same training parameter setting conditions. The accuracy and loss rate curves of each model training dataset and validation dataset are shown in Figure 8, and the recognition results after 6480 iterations are shown in Table 3. It is well known that the models that have undergone migration learning each have optimal architectures. The original result of wavelet time-frequency diagram is the pixel size of scale multiplied by the number of time points. Resize images to adapt to different network structures so that they can achieve the best results of learning. Resize does not change the accuracy of recognition, because CNN primarily extracts the energy distribution characteristics in time-frequency. We used the original input size of each model in order to ensure that the models retain their optimal architectures in this paper. The input size of AlexNet is 227 × 227 × 3, and others is 224 × 224 × 3.

The ResNet-50 model and the remaining four classical CNN models converge after 2000 iterations, as shown in Figure 8 and Table 3. The training accuracy and validation accuracy at convergence are above 90%, with ResNet-50 converging the fastest, MobileNet-v2 converging the second fastest, and AlexNet converging the slowest. The accuracy curves and loss value curves of both the training and validation datasets change more rapidly when the number of iterations is within 200. The change rate of the corresponding curve slows down when the number of iterations is between 200 and 2000. The curve basically stabilizes when the number of iterations is greater than 2000. The ResNet-50 model training accuracy rises to 97% from 65.6%, and the final model training accuracy reaches 99.61% when the number of iterations is between 200 and 450, which is shown in the ResNet-50 model in Figure 8. ResNet50 and MobileNetv2 are generally stable in terms of recognition stability except for fluctuations in a few positions in their recognition accuracy and loss value curves. While the other three are relatively poor, Alex and VGG-19 are more volatile in the early stage and converged slowly. The loss curve represents the fluctuation of the deviation between the predicted and true values of the model with the increasing of iterations number. The accuracy of the model is higher when the loss value is smaller, and the probability of error in prediction is therefore smaller. As shown in Figure 8c,d, the final training loss value of the ResNet-50 model is 0.0033 and the validation loss value is 0.0123. Compared with the other four models, ResNet-50 has the smallest loss value and the first convergence, while AlexNet has the largest loss value and the slowest convergence speed. Running time is also an important metric for the model performance evaluation. The shortest running time for AlexNet model was about 0.5 h and the longest for VGG19 model was about 1.26 h, however, the model in this study was 1.1 h and MobileNet was 1.23 h. Thus, the ResNet-50 model performed better than other models in recognition accuracy and stability. Features are the starting point of many computer image analysis algorithms and, therefore, the outstanding performance of ResNet-50 also reflects its excellent nonlinear feature extraction capability.

### 4.2. Comparison of the Dataset Size on Identification Performance

Nonlinear feature extraction relies on images, while the source of images is various datasets, and the datasets largely determine the recognition performance of the model. In order to study the effect of dataset size on the recognition results of multi-bolt loosening and preload force changing, we set five different sizes of datasets for model training. Because the number of samples obtained from the vibration generator test was greater, 100, 1000, 1500, 2500, and 4500 images, were randomly selected from each category of this test to form a new dataset, denoted as 100, 1000, 1500, 2500, and 4500, respectively. The accuracy of the models trained using five different size datasets is shown in Table 4. It is obvious that the model accuracy increases with the increase of the dataset. The validation accuracy and test accuracy of the model obtained after training on different size datasets range from 95% to 99.95% and 90.31% to 99.94%, respectively. Even with only 100 images per class of samples, the ResNet-50 model still has more than 95% test accuracy. This indicates that the CNN model with transfer learning also has good recognition performance for small sample datasets. However, in order to ensure that the model has higher accuracy and generalization capability, we still require sufficient image samples to ensure that the model can learn enough subtle nonlinear features to distinguish similar nonlinear features. The variation in verification accuracy and test accuracy is small when the number of samples is higher than 500. As shown in Table 3, the training accuracy and verification accuracy are 99.61% and 98.86% when the number of samples used in the hammer test is 500. In summary, when the number of each classification sample is only 500, the CNN model with ResNet-50 can achieve a remarkable recognition effect, and the recognition result is stable and has strong generalization ability.

### 4.3. Comparison of VAM Excitations on Identification Performance

**Figure 9:**
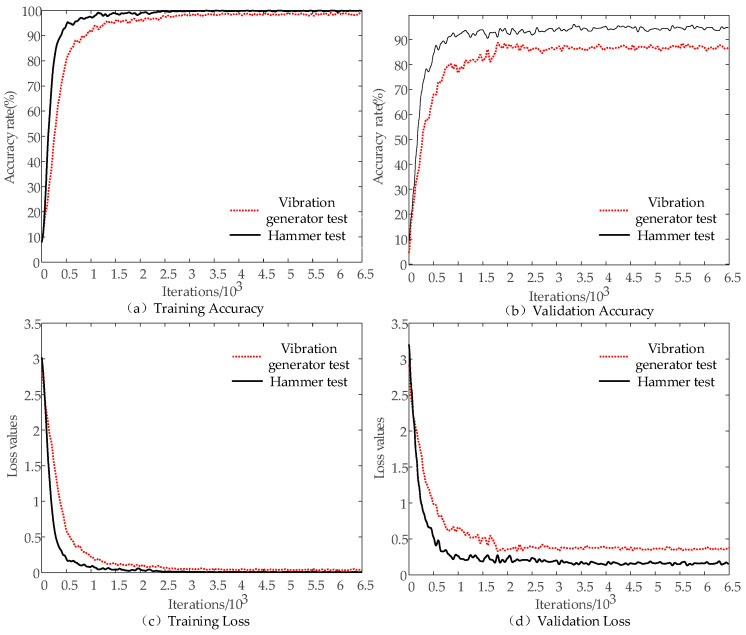
Comparison of different excitation forms on model performance of ResNet-50 model. (**a**) training accuracy; (**b**) validation accuracy; (**c**) training loss; and (**d**) validation loss.

The VAM method uses a combination of LF vibration and HF ultrasonic signals to achieve nondestructive identification, which can account for the local damage and overall damage. The effective combination of the two provides a new idea for the concealed damage of the multi-bolt connections. One of the most important nonlinear features of feature extraction is “repeatability”, meaning that the nonlinear features extracted from different images of the same scene should be the same. The vibration generator and hammer were used as LF excitation for VAM experiments in order to extract more accurate nonlinear features in this study. Moreover, other conditions were kept constant to obtain different time-frequency sample diagrams. The model identification results are shown in Figure 9.

Figure 9 shows the training models for the samples obtained from both tests converge after 2000 iterations, where the training and validation accuracies exceed 98% at convergence. It notes that the hammer test sample has the fastest convergence rate. When the number of iterations is less than 200, the accuracy curve and loss curve of the training dataset and the validation dataset change rapidly. The corresponding curve changes at a significantly slower rate when the number of iterations is between 200 and 1000. The curve tends to stabilize when the number of iterations is greater than 1000. As shown in the training model of the samples obtained from the hammer test in Figure 9, the training accuracy rises to 96% from 76.2% when the number of iterations is between 200 and 500. The final model training accuracy reaches 99.48% with less fluctuation. Its recognition accuracy and loss value curves are generally stable. The validation accuracy of the model is finally 96.24%, while the training loss and validation loss are 0.0042 and 0.0283, respectively. The changing trend of the training model of vibration generator test sample is similar to that in the hammer test, but each accuracy and loss value of vibration generator test is lower compared to that in the hammer test. However, the training and validation accuracies of vibration generator test are still 98.2% and 87.4%, respectively, and the training and validation losses are 0.0016 and 0.0297. This indicates that the force hammer as a form of LF excitation enables the ResNet-50 CNN model to perform the best image classification. Notably, the combination of the ResNet-50 model with linear FM can also provide some help in practical identification when the vibration generator is used as a form of LF excitation. It is observed that the accuracies of both models exceeded 98%, demonstrating the ResNet-50 model has a great capability to extract different image nonlinear features.

### 4.4. Verification of the Proposed CNN Model and VAM Excitation

**Figure 10:**
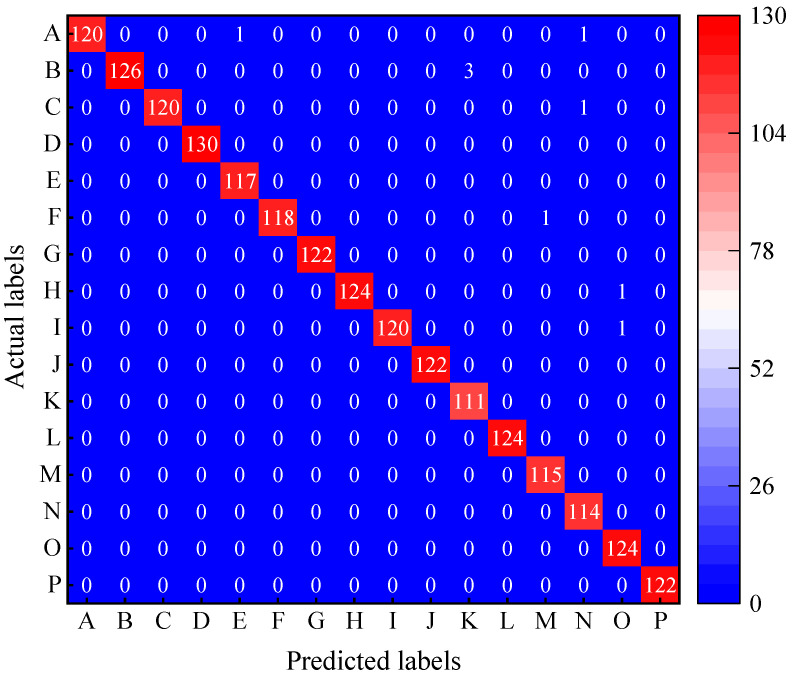
Confusion matrix.

A new test dataset was applied to test the model to check the effectiveness and generalization ability of the ResNet-50 model obtained after the above hammer test training. The test dataset was constructed as shown in Section 2.2. Four metrics were used to measure the classification accuracy of the model for the test samples in this paper, which were Confusion matrix, Precision, Recall, and Specificity [13]. The analysis results are shown in Figure 10 and Table 5.

The trained ResNet-50 model has a 99.5% classification accuracy and only a 0.5% error rate for the test samples in Figure 10 and Table 5. Although label E and M have one sample, N and O have two samples, and K has three samples that were wrongly classified into other categories, all its test samples were correctly identified and classified. The precision of Label K is 0.974 because three of its samples were misclassified to B, but its recall and specificity are 1. This also leads to a recall and specificity of only 0.977 and 0.998 for label B. However, as all samples corresponding to label B were accurately identified, its precision is still 1, and other results are similar.

The results indicate that the trained ResNet-50 CNN model is good at identifying different locations and different numbers of high-strength bolt loosening VAM signals under a variety of experimental conditions.

## 5. Conclusions

The bolt loosening during operation can reduce the load-carrying capacity, safety, and durability of the structures. In order to identify loosening damages in multi-bolt connections of large-scale civil engineering structures, we proposed a multi-bolt loosening identification method based on time-frequency diagrams and ResNet-50 CNN using VAM signals. Continuous wavelet transform was employed to obtain the time-frequency diagrams of VAM signals. After that, the CNN model was trained to extract damage-related nonlinear features from the raw time-frequency diagrams intelligently. This method helps to get rid of the dependence on traditional manual selection of the simplex and ineffective damage index and to eliminate the influence of the operational noise of structures on the identification accuracy.

Laboratory tests were carried out on a bolted connection specimen with four high-strength bolts of different degrees of loosening. The effects of different excitations, CNN models and dataset sizes were investigated. It could be observed that the ResNet-50 CNN model taking time-frequency diagrams of the hammer exciting VAM signals as the input showed the best performance in identifying the loosened bolts of various degrees of loosening at different positions. The training accuracy rose to 96% from 76.2% when the number of iterations was between 200 and 500. The final model training accuracy reached 99.48%, and the final model training accuracy of the vibration generator test was 98.2%. The results indicate that the proposed multi-bolt loosening identification method based on VAM and ResNet-50 CNN can identify bolt loosening with a reasonable accuracy, computational efficiency, and robustness. Moreover, it has low requirements for the size of the dataset, strong generalization ability, and accurate nonlinear feature extraction.

The method not only has high identification accuracy and stability, but also has low requirements for the test environment. It is easy to be applied to mobile devices and harsh engineering environments, and provides a new idea for online damage identification of structures in complex environments. Furthermore, only two cases (tightened or not) of each bolt were considered in this paper, and high-strength multi-bolt specimens with different preload forces and under more environmental coupling will be considered for testing in further work. This study only realizes the feasibility of the method from the principle, and the influence of the boundary conditions will be studied in the subsequent engineering practical applications.

## Figures and Tables

**Table 1:** Bolt-loosening cases.

Case	Bolt Number and State			
	Bolt I	Bolt II	Bolt III	Bolt IV
A	Loosening	Loosening	Loosening	Loosening
B	Loosening	Loosening	Loosening	Tightness
C	Loosening	Loosening	Tightness	Tightness
D	Loosening	Loosening	Tightness	Loosening
E	Loosening	Tightness	Tightness	Tightness
F	Loosening	Tightness	Tightness	Loosening
G	Loosening	Tightness	Loosening	Loosening
H	Loosening	Tightness	Loosening	Tightness
I	Tightness	Loosening	Tightness	Tightness
J	Tightness	Tightness	Tightness	Tightness
K	Tightness	Tightness	Tightness	Loosening
L	Tightness	Tightness	Loosening	Loosening
M	Tightness	Loosening	Loosening	Loosening
N	Tightness	Tightness	Loosening	Tightness
O	Tightness	Loosening	Tightness	Loosening
P	Tightness	Loosening	Loosening	Tightness

**Table 2:** Computer platform and environment configuration.

Software and Hardware Platform	Model Parameters
Operating system	Windows 10 a 64-bit system
CPU	AMD Ryzen Threadripper 2990WX 32-Core Processor
GPU	NVIDA Geforce RTX 2080 Ti
Memory	128G
Programmed environment	Matlab 2021a 64-bit

**Table 3:** Classification accuracy and loss of each model.

Category	Accuracy Rate		Loss Rate	
	Training Dataset (%)	Validation Dataset (%)	Training Dataset	Validation Dataset
AlexNet	91.97	90.82	0.0267	0.1794
GoogLeNet	97.02	96.15	0.0038	0.0687
MobileNet-v2	99.35	95.07	0.0063	0.0271
ResNet-50	99.61	98.86	0.0033	0.0123
VGG-19	99.48	96.48	0.0042	0.0283

**Table 4:** Validation and testing accuracy under different dataset sizes based on this model.

Dataset Size	Validation Accuracy (%)	Testing Accuracy (%)
100	95.00	90.31
500	98.02	98.04
1000	99.76	99.84
1500	99.81	99.79
2500	99.82	99.90
4000	99.95	99.94

**Table 5:** Precision, recall and specificity.

Label	Precision	Recall	Specificity
A	1.000	0.984	0.999
B	1.000	0.977	0.998
C	1.000	0.992	0.999
D	1.000	1.000	1.000
E	0.992	1.000	1.000
F	1.000	1.000	1.000
G	1.000	0.992	0.999
H	1.000	1.000	1.000
I	1.000	0.992	0.999
J	1.000	0.992	0.999
K	0.974	1.000	1.000
L	1.000	1.000	1.000
M	0.991	1.000	1.000
N	0.983	1.000	1.000
O	0.984	1.000	1.000
P	1.000	1.000	1.000

## Data Availability

Not applicable.
